# Policy assessment and policy development for physical activity promotion: results of an exploratory intervention study in 15 European Nations

**DOI:** 10.1186/1478-4505-10-14

**Published:** 2012-04-18

**Authors:** Alfred Rütten, Karim Abu-Omar, Peter Gelius, Susie Dinan-Young, Kerstin Frändin, Marijke Hopman-Rock, Archie Young

**Affiliations:** 1 Institute of Sport Science and Sport, University of Erlangen-Nuremberg, Gebbertstraße 123b, 91058 Erlangen, Germany; 2 Royal Free and University College London School of Medicine and University of Derby, Derby, UK; 3 Karolinska Institutet, SE-171 77 Stockholm, Sweden; 4 VU University Medical Center, PO Box 7057, 1007 MB Amsterdam, the Netherlands; 5 University of Edinburgh, Old College, South Bridge Edinburgh EH8 9YL, Edinburgh, UK

**Keywords:** Physical activity, Policy assessment, Policy development, Theoretical model, Questionnaire, Older people

## Abstract

**Background:**

Purpose of the study was to test a theoretical model to assess and develop policies for the promotion of physical activity among older people as part of an international intervention study.

**Methods:**

248 semi-standardized interviews with policy-makers were conducted in 15 European nations. The questionnaire assessed policy-makers' perceptions of organizational goals, resources, obligations, as well as organizational, political and public opportunities in the area of physical activity promotion among older people. In order to develop policies, workshops with policy-makers were conducted. Workshop outputs and outcomes were assessed for four nations nine months after the workshops.

**Results:**

Policy assessment: Results of the policy assessment were diverse across nations and policy sectors. For example, organizational goals regarding actions for physical activity promotion were perceived as being most favorably by the sports sector. Organizational obligations for the development of such policies were perceived as being most favorably by the health sector.

Policy development: The workshops resulted in different outputs: a national intersectoral action plan (United Kingdom), a national alliance (Sweden), an integrated policy (the Netherlands), and a continuing dialogue (Germany).

**Conclusions:**

Theory-driven policy assessment and policy-maker workshops might be an important means of scientific engagement in policy development for health promotion.

## Background

The World Health Organisation (WHO) states that health policies "arise from a systematic process of building support for public health action that draws upon available evidence, integrated with community preferences, political realities and resource availability" [1]. However, attempts of scientists to engage policy-makers in the development of health policies have been described as dancing with a "whirling dervish" and sometimes as failing altogether [2,3]. This article sets out to report on a European research project that attempted to assess and develop evidence-based, organizational policies for the promotion of physical activity among older people.

Difficulties of scientific engagement in processes of policy development may be rooted partly in the perception that research utilization and policy development by policy-makers are linear processes. Within such thinking, policy-making evolves from (1) evidence generated by scientists via (2) knowledge brokering to policy-makers to (3) action taken by policy-makers based on the evidence presented [4]. Others have suggested that policy development is rather a non-linear or even chaotic process [5,6]. Yet again, other studies on policy development in health promotion may suggest that determinants of policy development other than scientific evidence should also be considered. These include, for example, existing competences, value orientations that favor health [7], organizational self-interests [8], viability of adequate resources [9], persuasiveness of the policy idea, and political climate [10].

A number of concepts have been presented to guide scientific engagement in processes of policy development for health promotion. The WHO has developed an assessment instrument covering, among others, existing policies and legislative frameworks, the organization of health services, available human resources, links with other policy sectors, and research capacities to guide policy development for mental health care [11]. Another model for policy assessment featuring characteristics such as leadership and governance, service delivery, available financial resources, and available human resources has been proposed for accelerating policy development in the area of maternal and child care [12].

There have been only a few attempts at cross-national policy assessment and policy development for physical activity promotion. But cross-national concepts are of special importance since they might generate knowledge sensitive to differences in the political and welfare systems of nations. This kind of knowledge is especially important for effective policy development in relation to physical activity: While recent years have seen remarkable progress in the development of supranational [13-16] and national policies for physical activity promotion (23 out of 33 European Nations have at least one national policy document regarding physical activity and public health [17]), most of these policies have been described as being inconsistent and as lacking systematic implementation and evaluation.

This article reports on results of a project that assessed organizational policies for physical activity promotion among older people, and utilized the results to assist in the development of such policies in each of the contributing fifteen European nations. The project assessed organizational policies by a theoretical model that attempts to explain policy outputs and outcomes by organizational goals, resources, obligations, as well as organizational, political and public opportunities. In the view of this model, the assessment of these determinants can serve as a starting point for scientists to engage in a discourse with organizations in order to increase their policy output or outcome. For example, if the policy assessment yields that an organization might have sufficient resources, a high degree of obligations, and some opportunities to formulate actions on physical activity promotion, but lacks specific goals, scientists could assist such an organization to formulate such goals in order to increase the organizational policy output and outcome.

Going beyond most of the established models for research utilization as they have been outlined by Weiss et al. [5], our model provides clues on how to improve research utilization by working on the receptiveness of organizations rather than working solely on appropriately framing researcher's messages (as proposed e.g. by Landry et al., 2001) [18].

We believe that there are several points underlining the relative advantage of our framework of policy analysis and policy development based on von Wrights action theory as against others [19]: First, our model is theory-driven as it both identifies a set of major "causal drivers" (determinants) that influence policy-making and describes the mechanisms based on which these factors interact (logic of events) to influence policy impact (output and outcome). Second, it is more than just a theoretical aid to help us conceptualize reality in our minds: Its operationalization, both quantitative and qualitative, allows us to measure the determinants and to test the model as a whole. Empirical analysis and application has shown that the model actually works. Third, another advantage of the model is its parsimony. The limitation to just four determinants ensures that the model is easy to use and can be applied not only by scientists but also by practitioners. Fourth, due to its simplicity, the model may be used for cross-national comparisons or development efforts, as the four determinants can be assumed to operate under a broad range of political and societal environments.

### Theoretical model

The conceptual framework of this study was developed and tested in a six-nation project that transferred von Wright's [20] individualistic action theory to health policy analysis [21-23]. Von Wright's theoretical model explains individual behavior with four "determinants": wants, abilities, duties, and opportunities. The project adopted this model to explain organizational health related policies: Organizational goals (wants) refer to formally specified objectives of health policy actions, organizational resources (abilities) describe internal capacities for accomplishing health policy goals (e.g. personnel), organizational obligations are formal (e.g. treaties) or informal (e.g. organizational commitments) duties, organizational opportunities refer to internal (e.g. organizational changes) or political opportunities (e.g. changes in political climate, public or media interest) for organizations. The model has also been applied to other contexts [24,25] and is regarded as a relevant tool for describing the influence of research on health policy-making [26].

The WHO defines health policy as a "formal statement or procedure within institutions (notably government) which defines priorities and the parameters for action in response to health needs, available resources and other political pressures" [1]. Our approach, by contrast, is based on a broader definition of policy, which also includes informal institutional arrangements and procedures as well as rationales for action on health-related issues. From this point of view, policy analysis does not only include the investigation of documents and processes but also the assessment of capacities and resources for policy-making measured via the above-mentioned determinants. Policy development, in turn, not only comprises the formulation of policy documents or the adaption of procedures but also the improvement of capacities and resources. This may be achieved by working on policy determinants which policy analysis has shown to be underdeveloped.

The theoretical model applied in this study is not inextricably linked to one of the major theoretical frameworks on the policy process [27] but in our opinion the model considers fundamental components of such frameworks. For example, there are certain similarities between our model and Institutional Rational Choice (IRC) theory and the related framework of Institutional Analysis and Development (IAD) [28-30]. Both assume a certain degree of rationality and predictability of actions, and both allow for actors to be either individual or collective. Moreover, although not always congruent in their respective meanings, there are overlaps with regard to central categories (rules/obligations, motives/goals, and resources as central in both approaches). There are also certain links with the "Multiple Stream" (MS) approach [31,32]. For example, the key concept of "political windows" of opportunities in the MS-framework [31], p.173 overlaps with "political opportunities" and "public opportunities" in our model.

While IRC/IAD and MS are theoretically highly elaborated and accepted as major theoretical frameworks of the policy process, the model suggested here is rather simple. However, generalizability of "grand theory" may imply a number of limitations with respect to its practical applicability. Due to its very character as a framework many of IRC/IAD and MS concepts remain comparatively general and unspecific, making it somewhat difficult for practitioners to apply and operationalize them. Although various attempts have been made to improve their practical applicability, they do not lend themselves easily to being used for policy development. For example, in a recent paper a "multiple governance framework" have been suggested to provide for concepts influenced by IRC/IAD "which may assist low-or middle range theory formation and systematic empirical research" [33], p.558. However, while in Hill & Hupe's paper "application" mainly refers to the interpretation of selected data from two case examples of UK-policy in order to "illustrate" the "framework, and set out some of the issues it helps to highlight" [33], p.564, our theoretical model is more comprehensive in it's application. As illustrated in the present paper, it already can be used as framework for (1) data collection, (2) cross-national data analysis and, (3) policy development.

## Methods

Data was gathered in the context of the "European Network for Action on Ageing and Physical Activity (EUNAAPA)" project. EUNAAPA is a Europe-wide network of experts, scientists, and policy-makers that seeks to promote health, wellbeing and independence among older people through evidence-based physical activity. From 2006 to 2008, the Directorate General for Health and Consumers (DG SANCO) of the European Commission funded the establishment and initial activities of EUNAAPA. 21 institutions from 16 European nations (Austria, Belgium, Czech Republic, Denmark, Finland, France, Germany, Greece, Italy, the Netherlands, Norway, Poland, Portugal, Spain, Sweden, and the United Kingdom) took part in the project. EUNAAPA focused on (a) identifying effective instruments for assessing physical activity and physical functioning among older people, (b) critically comparing reputedly successful physical activity programs and promotion strategies with evidence-based best practice guidelines, and (c) disseminating the findings among policy-makers and contributing to their implementation.

### Data collection for policy assessment

An expert survey to assess the perception of organizational health policies for physical activity promotion among older people was conducted in all participating nations except the Czech Republic. In order to allow for structural equivalence in the comparative inquiry across nations [34], a sampling matrix was developed to distinguish between policy sectors (sports, health, social welfare) and levels of governance (national/regional, local). Each nation was requested to recruit at least one respondent from each cell of the sampling matrix. Relevant policy-makers were identified either through an expert rating or a snowballing referral system. Policy-makers were then approached either personally or by phone and informed about the purpose of the survey and the EUNAAPA project. In the UK, policy makers who had accepted the invitation to participate in a workshop completed the policy assessment questionnaire prior to the workshop. The standardized questionnaire was administered either by mail or by phone, depending on policy-makers' preferences. If necessary, a number of contact attempts and reminder calls were made.

### Instrument for policy assessment

The questionnaire used to assess organizational health promotion policies for physical activity promotion among older people was initially developed and tested in the MAREPS project [21]. For the present study, a short 14-item version of the original questionnaire, based on results of dimension reduction analysis [35], was utilized. Policy-makers were asked to report on the policy rationales of their respective organization for the promotion of physical activity among older people along the determinants of goals, obligations, resources, and opportunities. Goals and obligations were assessed with three items each, resources and opportunities with four items each (see Appendix A). For the items on goals, obligations, and resources, answer categories ranged from 1 (not true at all) to 5 (definitely true) on a 5-point Likert scale. Answer categories for opportunities ranged from 1 (situation has worsened) to 5 (situation has improved). Policy sector and policy level of respondents were assessed in the introductory part of the survey.

### Statistical analysis

For the descriptive analysis, we aggregated the responses for all dimensions of each determinant. Respondents were coded as having a favorable perception of a policy determinant if they answered "true" or "definitely true". From this, the mean percentages of respondents with positive perceptions of determinants were calculated by nation, or by policy sector or policy level. Due to the small sample sizes, within nations, results could not be analyzed by policy sector or policy level. The results section below gives an overview of the results from the 15 participating nations. Due to space restrictions, however, detailed results will only be reported in this article for the United Kingdom, the Netherlands, Sweden and Germany.

### Utilization of policy assessment data for policy development

Policy assessment questions and data were utilized to prepare national level workshops intended to increase the policy output and outcome for the promotion of physical activity among older people. A list of guiding questions and topics worth investigating was prepared for each national workshop. Invitations for the workshops were then sent to the policy-makers who had participated in the policy assessment survey.

At the workshops, the national EUNAAPA researchers first informed the policy-makers about project results for their nation and for Europe. In particular, researchers attempted to stimulate discussions on determinants that had been rated less favorably in the policy assessment across the different organizations within one nation. In these discussions, researchers would attempt to make organizations work on the determinants, that they had rated less favorably. The discussions at the workshops were recorded by workshop minutes and summarized in national reports. In addition, the project coordinator employed a written open-ended questionnaire to inquire senior researchers in four select countries (United Kingdom, the Netherlands, Sweden, and Germany) about their perception of the outcomes of the workshops. This follow-up survey was conducted nine months after the workshops.

## Results

### Cross-national policy assessment

Overall, 248 interviews with policy-makers were conducted in the 15 participating nations (see Table 1). Realized total sample sizes and sample sizes by policy sector varied starkly between nations. Between eight (Poland) and 34 (Netherlands) policy-makers were interviewed in each participating nation. Due to structural and organizational differences in some nations, the policy sector could not be determined precisely for some respondents.

**Table 1:** Description of the Sample by Policy Sector, EUNAAPA Survey

*Nation*	*N*	*n Sector Sport*	*n Sector Health*	*n Sector Social Care*	*Missing*
Belgium	15	9	1	5	

Denmark	30	5	14	8	3

Germany	11	4	3	4	

Greece	10	8	1	1	

Spain	16	6	5	5	

Finland	12	5	5	2	

France	16	8	3	5	

Italy	16	2	9	5	

Netherlands	34	13	13	8	

Austria	9	4	1	3	1

Portugal	12	7	2	3	

Sweden	23	6	8	9	

United Kingdom	17	6	6	3	2

Poland	8	1	1	1	5

Norway	19	4	12	3	

*Total*	*248*	*88*	*84*	*65*	*11*

**Figure 1:**
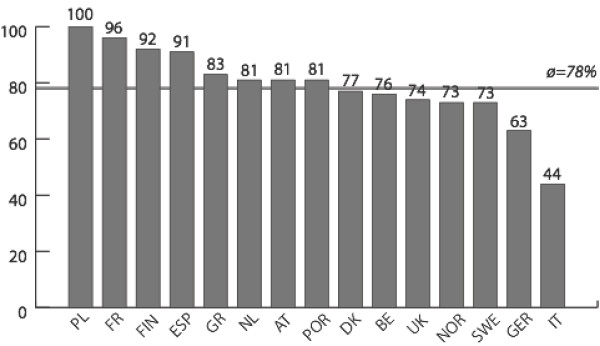
**Goals**. Mean percentage of respondents with a positive assessment of goals related to physical activity and ageing, by nation.

**Figure 2:**
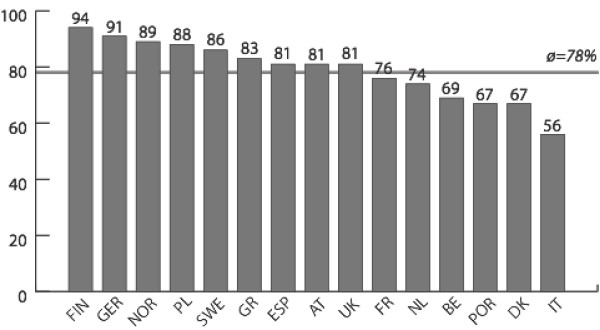
**Obligations**. Mean percentage of respondents with a positive assessment of obligations related to physical activity and ageing, by nation.

**Figure 3:**
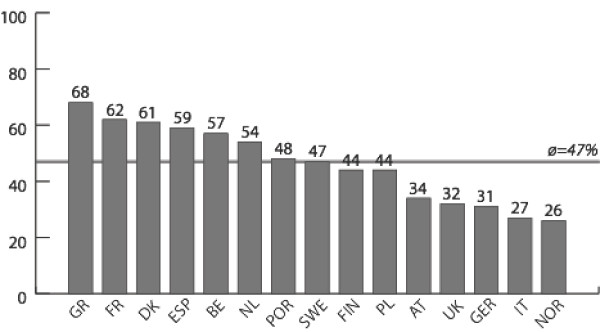
**Resources**. Mean percentage of respondents with a positive assessment of resources related to physical activity and ageing, by nation.

**Figure 4:**
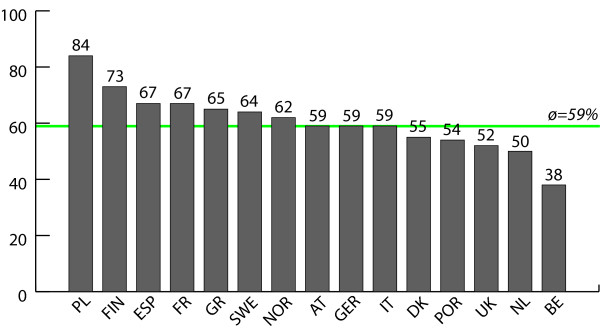
**Opportunities**. Mean percentage of respondents with a positive assessment of opportunities related to physical activity and ageing, by nation.

Figures 1, 2, 3, 4 report policy-makers' perceptions of organizational policy determinants by nation. The mean percentage of respondents with a positive perception of organizational *goals *in the area of physical activity promotion among older people was highest in Poland (where 100% of respondents rated organizational policy goals as being concrete, officially spelled out, and focused on improving the health of the population) and lowest in Italy (where only 44% of respondents had a positive perception of goals). Regarding *obligations*, the most positive perceptions were found in Finland (94%), and least positive perceptions were found in Italy (56%). Across nations, *resources *were rated least favorably compared to the other determinants. In Norway, 26% of respondents had a positive perception of resources, and in Greece this percentage was 68%. While only 38% of respondents in Belgium believed that *opportunities *had improved during the last year, the share of positive responses was 84% in Poland.

Table 2 shows the mean percentages of policy-makers having favorable perceptions of organizational policy determinants, analyzed by policy sector and level. *Goals *were rated most favorably by policy-makers from the sport sector (83%), while *obligations *were rated most positively by policy-makers from the health sector (81%). Regarding sufficient *resources *to carry out policy actions, the sport sector had the most favorable perception (56%). There were no pronounced differences in the perception of determinants between the national and regional/local policy levels of policy-makers. *Opportunities *for policy action were rated slightly more favorably by respondents operating on the national level (61%).

**Table 2:** Mean Percentage by Policy Sector and Level of Perceptions of Policy-Makers on Policies for Ageing and Physical Activity according to the Theoretical Model, EUNAAPA Survey

*Percentage of respondents with a positive assessment of the respective determinant*				
***Policy Sector/Level***	***Goals***	***Obligations***	***Resources***	***Opportunities***

Sport	83	75	56	60

Health	73	81	41	60

Social Care	75	75	43	56

National	79	79	47	61

Regional/Local	77	75	48	56

### Case studies: detailed policy assessment

#### United Kingdom

The analysis for the UK showed a comparatively high level of obligations, a rather negative assessment of available resources, and goal definition problems for some organizations. In addition, researchers compared existing physical activity programs and promotion strategies with best practice guidelines. Four specific recommendations were put up for discussion at the national workshop: (1) Dedicated instructor qualifications and training routes for instructors working with older people, (2) benchmarking employer standards for instructors working with older people, (3) increasing access via local authority outreach teams, health services, the care home sector, and non-governmental organizations, and (4) more powerful promotion of physical activity to older people.

#### Sweden

The analysis for Sweden pointed to three potential topics for the national workshop. First, compared to other nations, policy-makers from some organizations reported that the goals of their organizations were not concrete enough and did not focus on improving the health of the population. Interestingly, these problems seemed to pertain almost exclusively to organizations at the national level. Second, many organizations seemed to lack both personnel and financial resources for activities related to physical activity promotion among older people. Again, the problem seemed to be more pressing at the national than on the local level. Finally, the Swedish survey included a comparatively large number of organizations which, despite reporting favorable policy determinants, were not active in the field of physical activity and ageing, calling for a discussion on how to integrate them into existing efforts by other players.

#### The Netherlands

Overall, survey results from the Netherlands were comparatively positive. Nonetheless, two areas for potential improvement were identified for discussion at the policy-maker workshop. First, several local organizations seemed to fail to recognise that scientific evidence had driven the call for action in the field of physical activity promotion for older people. Second, while resources were generally assessed more positively than in other nations, all four items used in the survey indicated room for improvement. In particular, a considerable number of policy-makers reported a lack of support from the general public for actions related to physical activity promotion among older people.

#### Germany

For Germany, two major issues were identified in the policy assessment survey. First, about half of the policy-makers reported that their organizations had problems formulating or spelling out concrete goals. Second, while German policy-makers were among those with the most negative rating of resources in the entire survey (together with those from Italy and Norway), they also felt more obliged to become active than policy-makers in any other country except Finland. While the former finding was used at the workshop to initiate a discussion on the different perceptions of resources in European nations, the latter was emphasized by the EUNAAPA researchers to be a potential asset for future policy development in the field.

### Case studies: workshop outputs and outcomes

#### United Kingdom

31 policy-makers attended the workshop in the United Kingdom. The most tangible result was a comprehensive report of the workshop. The report summarized the workshop presentations and discussions, specified eight "action points", and designated individuals and/or organizations to take forward the agreed improvements to the promotion, provision, safety and effectiveness of physical activity opportunities for older people in the United Kingdom. Interagency collaboration was enhanced as a direct result of the workshop. For example, several key agencies from different policy sectors met for the first time at the workshop. Furthermore, collaborative efforts were more sharply focused as a result of the joint development of action points. Nine months after the workshop, one action point had been achieved, a second (a publication) was in the final stages of completion, and action on four further points was ongoing. In two other points, there had been no action.

#### Sweden

18 policy-makers attended the workshop in Sweden. It was decided to form a national alliance for physical activity promotion among older people to facilitate (1) physical activity friendly environments and (2) the development of local organizations providing fitness check-ups and tailored advice in relation to physical activity for older people. The alliance shall be authorized by the government to perform work in this area in the future. Policy-makers also agreed that national guidelines for different types of evidence-based physical training for older people should be developed in the future and that organizations dealing with older people should be provided with additional information on the benefits of physical activity in old age. Nine months after the workshop, some of the organizations involved continued to meet in order to develop capacities for physical activity promotion. A newly established funding program by the federal government helped the group to overcome initial financial resource problems and provided the opportunity to follow up on activities launched under the EUNAAPA project.

#### The Netherlands

The workshop was attended by 13 policy-makers. All in all, the information provided by researchers at the workshop was deemed to be valuable. Some tension was evident between policy-makers from organizations working at the local level and those at the national level. While policy-makers from the local level stressed that a sufficient 'good practice' base for physical activity promotion among older people already existed, national level policy-makers called for greater recognition of the importance of evidence-based work. Nine months after the workshop, one outcome was the decision by some organizations to join forces for the implementation of an innovative local care system that would include physical activity stimulation among older people. Another outcome of the meeting was that organizations reached consensus about formulating an integrated policy on the topic. Key action points of this policy are the qualification of instructors and exercise prescription by medical practitioners. The integrated policy shall be developed by organizations from the health care sector and municipalities.

#### Germany

The workshop in Germany was attended by 23 policy-makers. Policy-makers generally confirmed the results of the policy assessment survey. The main focus of the discussion was on means to stimulate policy development. Most policy-makers agreed that the established organizations of the sport and health sector generally have sufficient resources for policy development. It was decided that no new board/network was needed for this purpose and that it would suffice for the established organizations in this field to continue working on the topic. However, some of these organizations invited EUNAAPA researchers to join their efforts. Nine months after the workshop, researchers remained engaged in a dialogue with a regional ministry on issues pertaining to physical activity promotion among older people. This dialogue resulted in the support of the ministry for another international project on the topic of physical activity and ageing. Other than that, no positive or negative outcomes can be reported.

## Discussion

This study has presented results of an effort to engage scientists in organizational policy assessment and policy development for physical activity promotion in fifteen nations. The concept employed featured (a) an assessment of organizational policies by means of a survey among policy-makers from relevant organizations and (b) engaging them in a discourse on based on the results of the policy assessment in order to increase their policy outputs and outcomes by a structured workshop in each nation.

The results of the survey indicate that determinants of policy outputs and outcomes for physical activity promotion among older people vary starkly among organizations in participating nations. In all nations, however, the determinant that policy-makers were most critical about was resources. While this might suggest that redirecting resources to physical activity promotion among older people would be an important means to increase policy outputs and outcomes, attempting to tackle this sensitive issue directly may prove to be futile. Alternatively, researchers might want to focus on improvements in the other three determinants of our model, thus indirectly achieving a shift of resources within organizations. For example, policy-makers in some nations reported a lack of concrete goals for policies on the issue of aging and physical activity. According to our theoretical model, researchers in these countries might be able to assist organizations in formulating such concrete goals. Not only would this stimulate policy-making, it might also prompt the organizations in question to provide more resources to reach their newly specified goals.

Meanwhile, policy-makers in a number of nations indicated that opportunities (e.g. media interest in the topic of ageing and physical activity) have improved in recent years. Again, according to the theoretical model, these improved opportunities are an important determinant of relevant policy outcomes.

Regarding the different policy sectors, policy-makers from the sport sector showed the most favorable perceptions of goals and resources. Consequently, the development of physical activity promotion policies might advance more rapidly if this sector is systematically engaged in such efforts. Respondents from the health sector, in turn, featured the most negative perceptions of available resources, while their perceptions of obligations were strongest compared to the other sectors. In order to stimulate policy development, it might thus be advisable to foster collaborations between the health and sport sectors for physical activity promotion among older people.

The case study results of the workshops in the United Kingdom, Sweden, the Netherlands, and Germany suggest that collecting expert survey data on policy determinants and presenting an analysis of such data at a workshop with policy-makers might enable researchers to engage in a discourse with relevant organizations on topics of health promotion. In all four nations, researchers observed at least some beneficial results. This included the continued collaboration with a regional ministry (Germany), the formulation of goals towards integrated policy-making (the Netherlands), the establishment of an alliance on the topic (Sweden), and the formulation of, and work on, a national action plan (United Kingdom).

However, it should be recognized that these results represent the perception of the researchers who were engaged in the project and thus might differ from the perceptions of policy-makers attending the workshop. In some nations, there is evidence that conflicts surfaced between the different policy-makers taking part in the workshops. This included, for example, discussions on current practice and its evidence-base between local and national policy-makers in the Netherlands. In Germany, organizations already functioning as interest brokers for physical activity promotion among older people were reluctant to agree on a way forward, potentially indicating competing interests in policy development. These results may be a further indication that processes in policy development for health promotion are non-linear.

Given the small number of cases compared, differing workshop results might also be explained by different value orientations of policy-makers and researchers in the four case study countries towards research utilization. Also, welfare-state orientations might play a role in shaping policy-makers' receptiveness for the issue of physical activity and ageing [36]. In this regard, workshop accomplishments in the Netherlands and Sweden might be attributed partly to the social-democratic welfare-state regimes in these nations.

We acknowledge that this study has a number of limitations and thus yields results that are explorative in nature only. For one, the sampling process of the survey was intended to achieve structural equivalence. However, in a number of nations, such equivalence has proven to be difficult to accomplish. This is due to stark differences within the organizations/sectors involved in physical activity promotion in the different nations. This problem reflects the general difficulties of cross-national comparative social inquiries [37]. Consequently, the analysis by sector may be rather incoherent in some cases. While the instrument for the assessment of policy rationales has already been tested successfully in a number of research projects, to infer that responses of an individual representative of an organization are representative of the organization as a whole (or, for that matter, of national public policies in general) remains a matter for debate.

Also, our results of the policy assessment may be biased due to the small sample sizes. These small sample sizes, for example, did not allow for a meaningful analysis of results by nation and policy level in numerous nations. It can, however, be assumed that in most nations policy determinants might systematically vary between organizations who are working on the local, regional, or national level.

It would also have been desirable to present a more comprehensive picture of organizational policies on the issue of aging and physical activity in the different nations. While some of this information were gathered as part of the survey, the presentation of results appeared difficult within the scope of this paper.

Some may criticize our approach of using national workshops to feed the results of our policy assessment into the process of policy development as being somewhat intuitive. Especially, since such an approach could be interpreted as being rather participatory. As a matter of fact, transforming quantitative survey data into a qualitative approach of policy development by the means of a workshop proved to be difficult for the EUNAAPA project.

In addition, the concrete effects of the presentation and discussion of policy assessment results on the results of the workshops is difficult to assess. The same applies to the actual impact of the workshops on policy development. The results reported in this article are preliminary in nature and thus only provide some very limited insights into the non-linear processes of policy development that may have taken place in the different nations. In some cases, results seemingly accomplished through the workshops may in fact have been only loosely related to these meetings. In part, these difficulties exemplify the general lack of understanding and availability of methodological tools for enhancing research utilization.

Beyond these methodological limitations, our attempt to increase the receptiveness of organizations for research utilization by focusing on means to alter the organizational context might be considered problematic, as it raises general questions about the science being value-free (*Wertfreiheit*) [38]. On the other hand, there have been calls for research to explicitly play an active role in shaping social action [39]. From this point of view, our approach seems to expand existing explanatory models of research utilization, offering the potential for enhanced scientific engagement in policy development. Based on our experience in this project, we would encourage the development of comprehensive theories and methods to initiate and evaluate scientific enterprises for the development of health promotion policies.

## Conclusions

This study has attempted to assess and develop policies for health promotion in the area of physical activity for older people on the organizational level. Since physical activity promotion is an intersectoral challenge, our approach may be useful to identify suitable starting points for research utilization and health promotion policy development involving different sectors. The WHO Global Strategy on Diet and Physical Activity and the corresponding framework for monitoring the implementation of physical activity promotion policies have paved the way for calls to synchronize cross-national efforts for policy development and implementation [13,40]. Concepts such as ours might help to guide researchers to engage policy-makers in these processes. Our theoretical model is applicable across nations, sectors and policy issues, it is parsimonious, and it has been validated in international studies. As such, it might not only prove to be valuable in effecting improved practice in physical activity promotion in the future, but it might also be applicable to some extent to policy development in other health promotion contexts.

## Competing interests

The authors declare that they have no competing interests.

## Authors' contributions

AR served as a principal investigator of the project, AR designed the data collection and analysis and drafted the manuscript. KAO and PG assisted in data collection, analysis, and drafting of the manuscript. SDY, KF, MHR, and AY conducted project work in their respective nations. They collected and analyzed national level data and provided integral comments on drafts of the manuscript. All authors read and approved the final manuscript.

## Appendix A. List of Items of the MAREPS Short-List for Policy Assessment in Health Promotion

### Goals

The goals are concrete enough

The goals are officially spelled out

The action is centered on improving the health of the population

### Obligations

Scientific results demand the action

The action is part of my professional duty

Personally, I feel obliged to do something in this field

### Resources

The population supports the action

There is enough personnel

My organization has the necessary capacities

There are sufficient financial resources

### Opportunities

The involvement of the population has worsened/improved

The media's interest has worsened/improved

My own involvement has worsened/improved

The cooperation within my organization has worsened/improved
